# Data on the yield and quality of organically hybrids of tropical tomato fruits at two stages of fruit maturation

**DOI:** 10.1016/j.dib.2019.104031

**Published:** 2019-05-30

**Authors:** Syariful Mubarok, Fathimah Fathinatunnabilah Farhah, Anas, Nursuhud Suwali, Dikdik Kurnia, Kusumiyati, Erni Suminar, Hiroshi Ezura

**Affiliations:** a Department of Agronomy, Faculty of Agriculture, Universitas Padjadjaran, Bandung, 45363, Indonesia; b Department of Chemistry, Faculty of Mathematics and Natural Sciences, Universitas Padjadjaran, Bandung, 45363, Indonesia; c Faculty of Life and Environmental Sciences, University of Tsukuba, Tsukuba, 305-8572, Japan

## Abstract

Organic and traditional cultivation techniques significantly affect the yield and quality of tomato fruit. To achieve the highest possible production of hybrid lines, the appropriate cultivation system is needed. The application of different cultivation systems was expected to improve the yield and fruit quality of three new tropical hybrid tomatoes varieties that prolong fruit shelf life. This experiment was conducted to identify the effect of the different cultivation systems on the yield and fruit quality of three hybrid tomatoes from different tropical parental backgrounds (‘Mutiara’, ‘Intan’ and ‘Ratna’). Those hybrid lines were cultivated with two farming systems (organic and conventional cultivation system), and the fruit quality was analysed at two stages of fruit maturation (Breaker and Red).

**Table:** Specifications Table

Subject area	*Agriculture*
More specific subject area	*Postharvest, Horticulture*
Type of data	*Figure*
How data was acquired	*Refractometer model 3810 Pal-1, Advanced Bench pH Meter 3510, AquaMate 8000 UV–Vis Spectrophotometer*
Data format	*Analysed*
Experimental factors	This experiment consisted of three factors (hybrid/F1 varieties, cultivation methods, and stage of fruit maturation) and was repeated four times. Three hybrid lines of tropical tomatoes varieties (Mutiara F1, Intan F1, and Ratna F1) were cultivated using two methods of cultivation (organic and conventional cultivation systems), and the fruits were harvested at two stages of fruit maturation (Breaker and Red) in triplicate.
Experimental features	*Determination of fruit yield, fruit quality i.e. fruit water content, titratable acidity, total soluble soild, pH, β-carotene and lycopene, and also data of micro-climate*
Data source location	*Sumedang, Indonesia.*
Data accessibility	*The data are obtainable within this article and publicly accessible.*
Related research article	Mubarok, S., Okabe, Y., Fukuda, N., Ariizumi, T., and Ezura, H. 2015. Potential use of a weak ethylene receptor mutant *Sletr1-2,* as breeding material to extend fruit shelf life of tomato. Journal of Agricultural and Food Chemistry. 63: 7995–8007 [1]. Vinha, A.F., Barreira, S.V., Costa, A.S., Alves, R.C., and Oliviera, M.B. 2014. Organic versus conventional tomatoes: influence on physicochemical parameters, bioactive compounds and sensorial attributes. Food and Chemical Toxicology. 67: 139–144 [2]. Makinde, A.I., Jokanola O.O., Adedeji J.A., Awogbade A.L., and Adekunle A.F. 2016. Impact of organic and inorganic fertilizers on the yield, lycopene, and some minerals in tomato (*Lycopersicum esculentum* Mill.) fruit. European Journal of Agriculture and Forestry Research. 4: 18–26 [3].

**Table:** 

**Value of the data** • The data obtained here will contribute to our understanding of the relationships between cultivation systems and changes in tomato quality. • The data could be used for practitioner and as basic data of further research.

## 1 Data

**Fig. 1:**
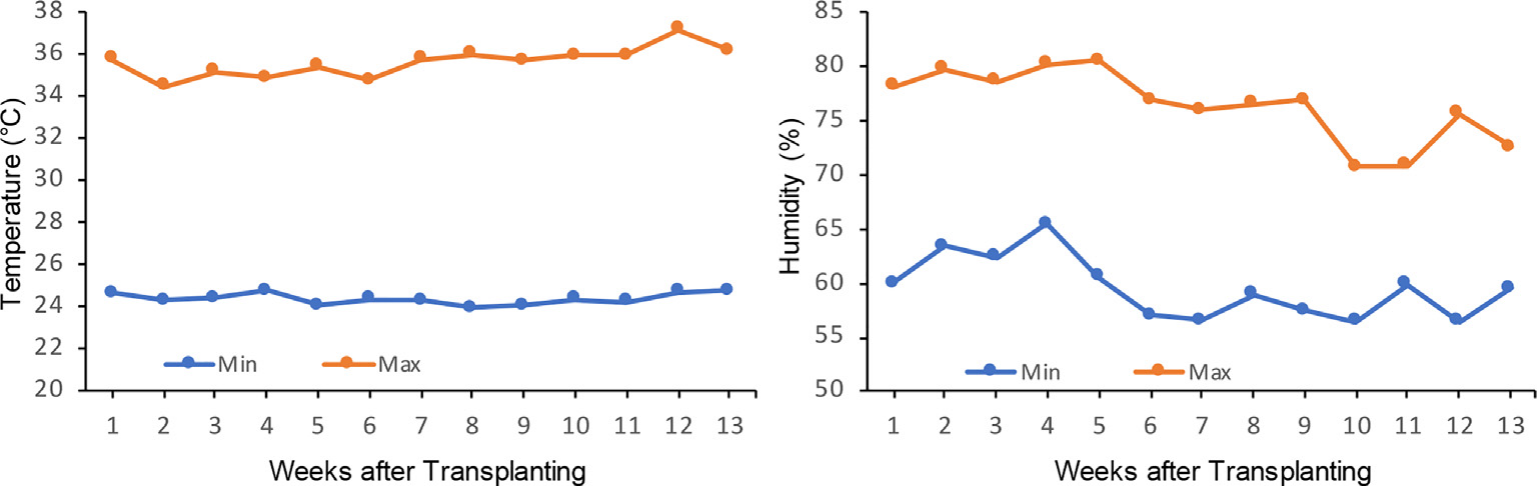
Micro-climate data (temperature and humidity) during three months of experimental period.

**Fig. 2:**
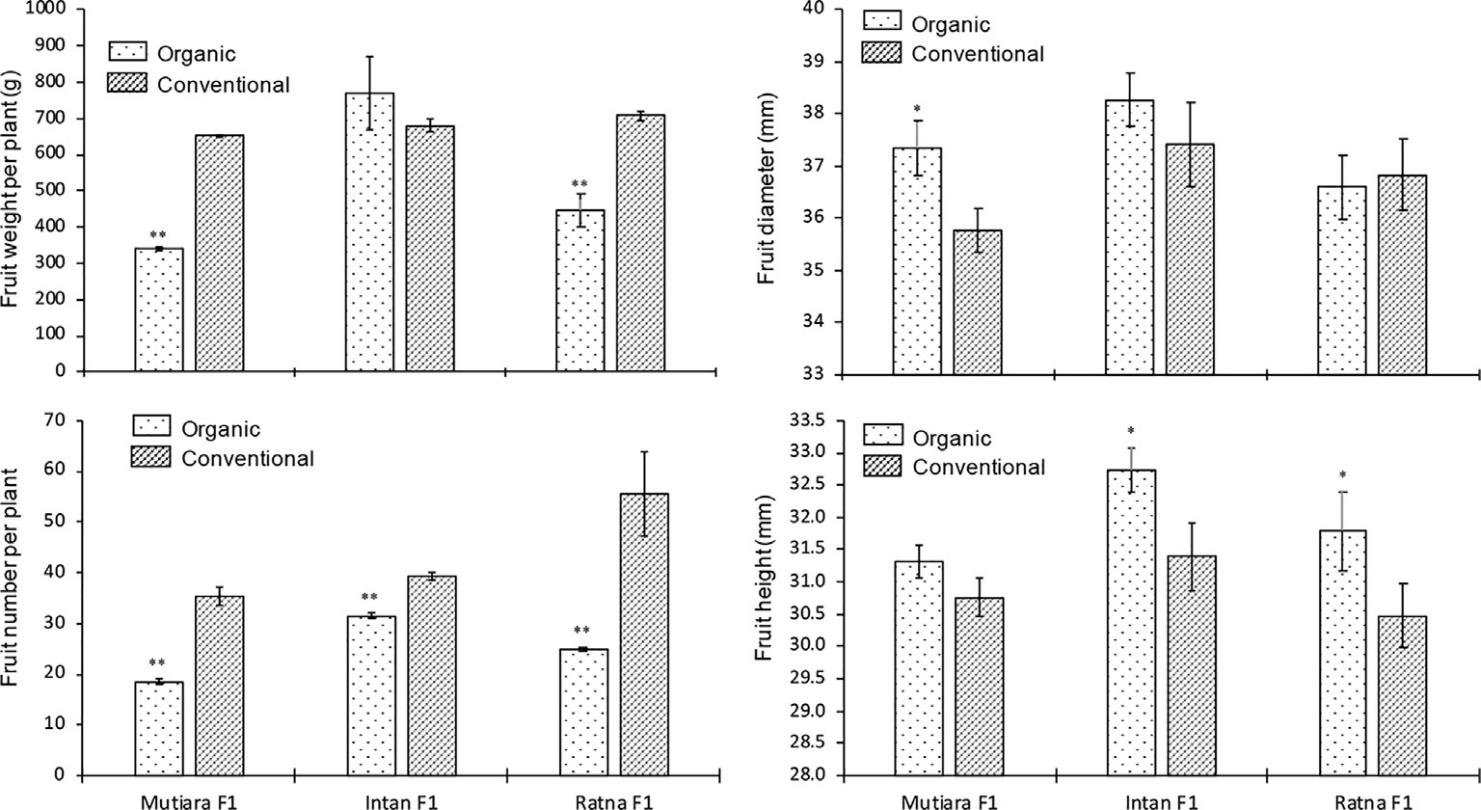
Fruit yield responses of tomato fruits from organic and conventional cultivation system. Mean ± Standard Error (SE, n = 4) followed by one and two asterisk are significantly different compare to conventional cultivation system in each hybrid line according to the Student's T-Test at p < 0,05 and p < 0,01, respectively.

**Fig. 3:**
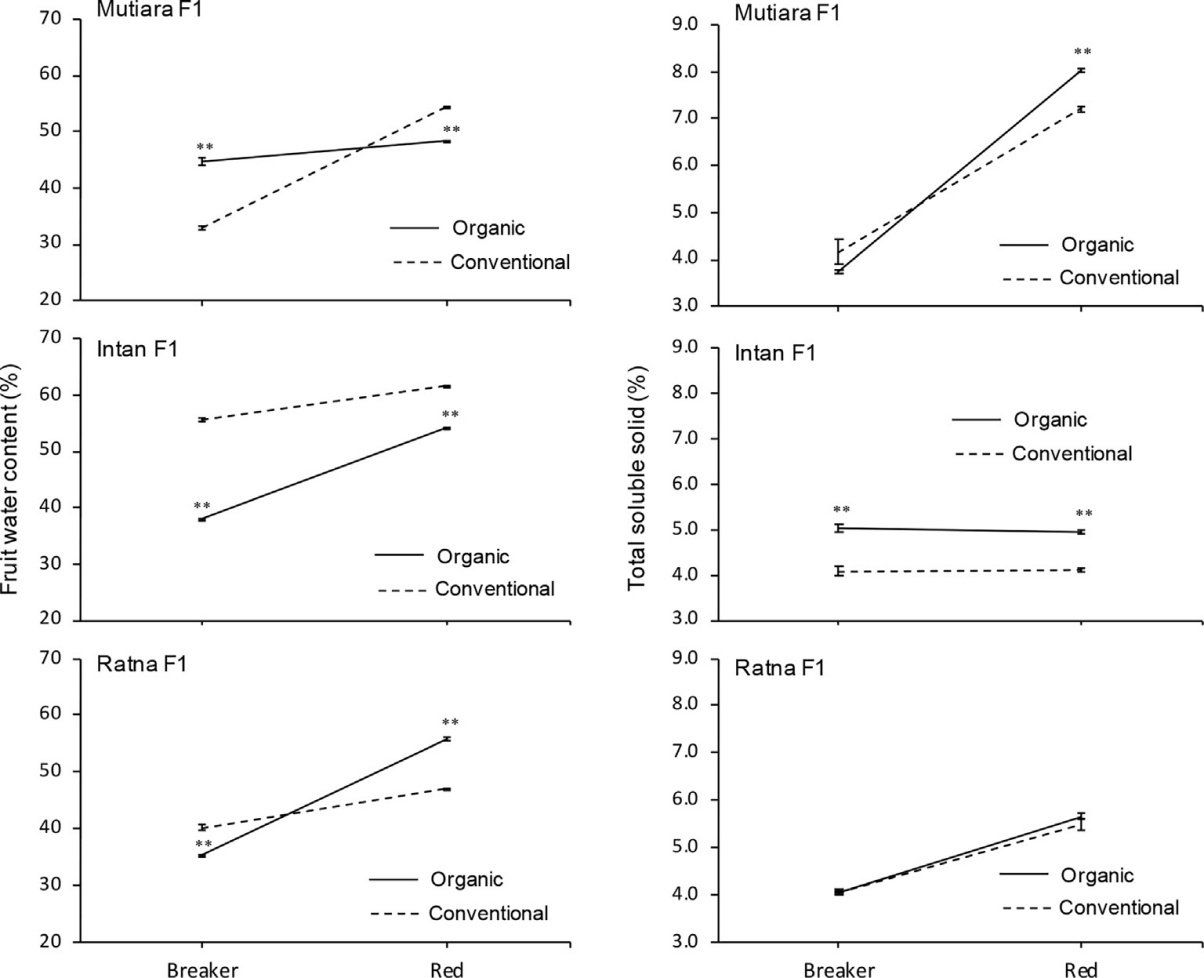
Fruit water content and total soluble solid (TSS) of two stages of fruit maturation from organic and conventional cultivation system. Mean ± Standard Error (SE, n = 4) followed by two asterisk are significantly different compare to conventional cultivation system in each hybrid line according to the Student's T-Test at p < 0,01.

**Fig. 4:**
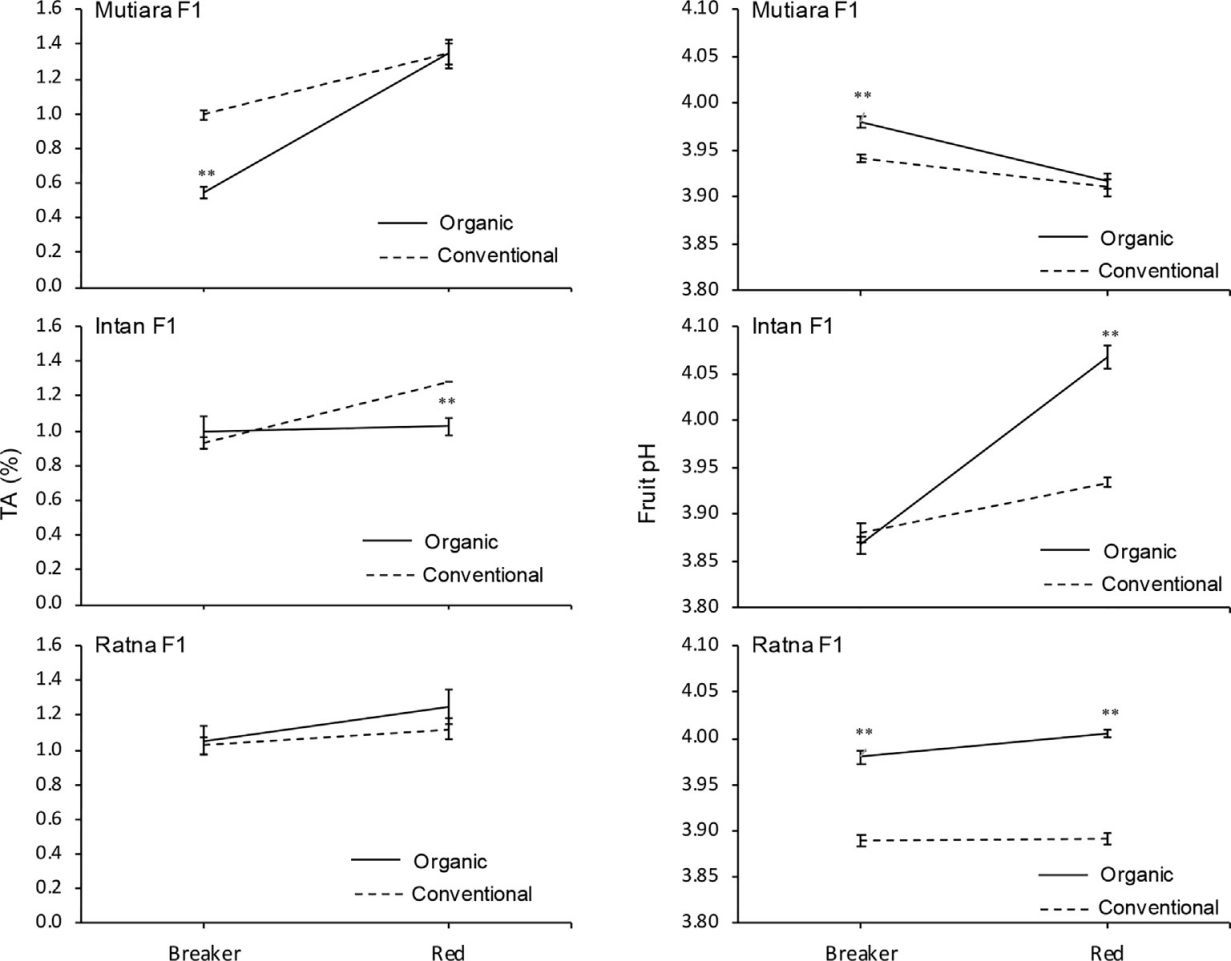
Fruit titratable acidity (TA) and pH of two stages of fruit maturation from organic and conventional cultivation system. Mean ± Standard Error (SE, n = 4) followed by two asterisk are significantly different compare to conventional cultivation system in each hybrid line according to the Student's T-Test at p < 0,01.

**Fig. 5:**
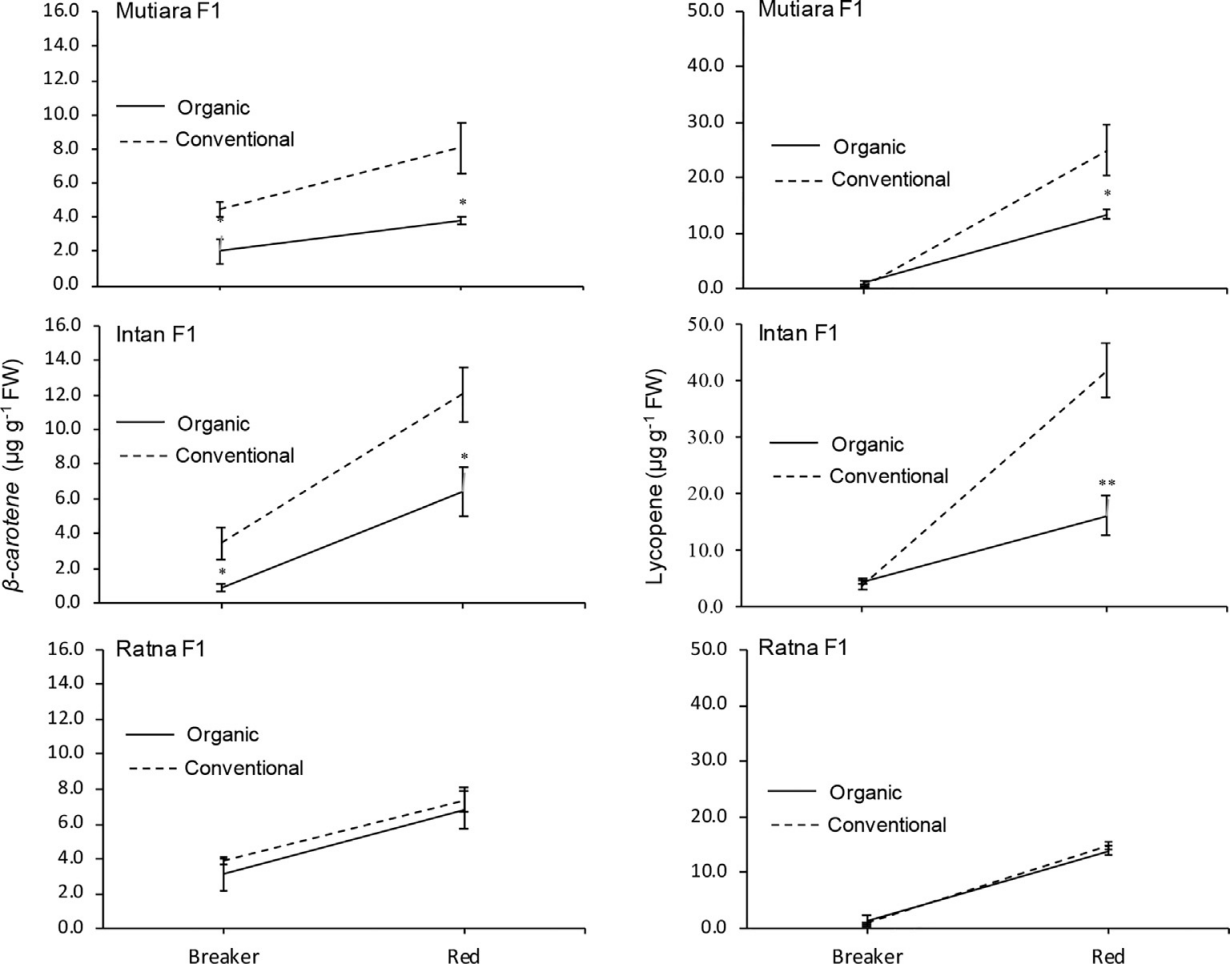
*β-carotene* and lycopene of two stages of fruit maturation from organic and conventional cultivation system. Mean ± Standard Error (SE, n = 4) followed by two asterisk are significantly different compare to conventional cultivation system in each hybrid line according to the Student's T-Test at p < 0,01.

The data report the fruit yield and quality of three hybrid tomato lines grown under two cultivation systems, organic and conventional. We analysed several parameters related to micro-climate and the fruit yield and quality (fruit weight per plant, fruit number per plant, fruit diameter, fruit weight, titratable acidity, fruit water content, total soluble solid, and fruit pH). Fig. 1 shows micro-climate data, and Fig. 2 shows the effects of two cultivation systems on fruit weight per plant, fruit diameter, fruit number per plant, and fruit length data. Fig. 3 shows data on fruit water content and total soluble solid (TSS). Fig. 4 reports data on fruit titratable acidity (TA) and pH. Fig. 5 shows data on *β-carotene* and lycopene.

## 2 Experimental design, materials, and methods

### 2.1 Plant preparation and cultivation

Three hybrid tomato cultivars (Mutiara F1, Intan F1, and Ratna F1) were cultivated on two cultivation systems (organic and conventional system) within a 100 m^2^ of tunnel type screen house at the Field of Research Center of Faculty of Agriculture, Universitas Padjadjaran, Indonesia from June 2018 to August 2018. The micro-climate condition of the greenhouse is crucial to the impact of the yield and fruit quality [4], therefore, during experimental period, air temperature and humidity were measured using an HTC-2 digital thermo-hygrometer (HTC Instruments, India). Seeds were sown in a seed tray at the beginning of the plants’ cultivation with soil and cocopeat (1:1/v:v) as a growing medium. After 3–4 weeks, the tomato plants were transplanted into a 30-cm polybag with a growing medium of compost and husk charcoal (1:1/v:v). The differences between organic and conventional cultivation system were in the used of organic or inorganic fertilizer and also pest/disease control. The fertilization was applied every week with 1 ml/L of liquid organic fertilizer for organic cultivation system and 1 g/L of NPK fertilizer for conventional cultivation system. No chemical fertilizer and pesticide were applied under the organic cultivation system. To analyse the fruit quality, the fruits were harvested at two stages of fruit maturation, namely Breaker (Br) and Red (Br+7).

### 2.2 Fruit yield analysis

The same fruit maturation stage on Br and Br+7 were harvested to be used for fruit yield and quality analyses. Several parameters related to fruit yield were analysed (i.e., fruit weight per plant, fruit diameter, fruit number per plant, and fruit length).

### 2.3 Analysis of the fruit water content

The fruit water content was analysed according to the method described by Aventi [5]. Briefly, fruit samples were dried using an oven with a temperature of 60 °C until they reached stable, dried fruit weight. Fruit water content was measured using the following equation:

$$\text{\%}\;\text{Fruit}\;\text{water}\;\text{content}=\frac{a-b}{b}\;\times \;100\%$$

a: fresh fruit weight (g)

b: dried fruit weight (g)

### 2.4 Analysis of the titratable acidity (TA), pH, and total soluble solid (TSS)

TA was measured using the modified titration method described by Mubarok et al. [6], [7]. Briefly, 5 g of fresh fruit was homogenised with 50 mL distilled water and then titrated up to pH 8.1 with 0.1 N sodium hydroxide. TA and pH were determined using an Advanced Bench pH Meter 3510 (Jenway, United Kingdom). TSS was used to estimate the fruit sugar content and measured with a refractometer model 3810 Pal-1 (ATAGO CO., LTD., Japan).

### 2.5 Analysis of β-carotene and lycopene

The β-carotene and lycopene contents were analysed at the breaker and red stages of fruit maturation, as done by Mubarok et al. [1] with some modification. Briefly, 7 mL of hexane/acetone (6:4 v/v) was used to extract the 700 mg tomato fruit pericarp. Then, the absorbance of the clear supernatant was measured using an AquaMate 8000 UV–Vis Spectrophotometer (Thermo Scientific, United States of America) at four absorbances namely 663 (A_663_), 645 (A_645_), 505 (A_505_), and 453 (A_453_). The following equations were used to measure lycopene (C_LYC_) and. *β*-carotene (C_CAR_):

C_LYC_ = −0.0458 (A_663_) + 0.204(A_645_) + 0.372(A_505_) – 0.0806(A_453_)

C_CAR_ = 0.216(A_663_) – 1.22(A_645_) – 0.304(A_505_) + 0.452(A_453_)

The content of lycopene and β-carotene were represented in micro-grams per grams of fruit fresh weight (μg/g FW).

### 2.6 Data analysis

This experiment was carried out with four replicates in a randomised block design. The data were represented as the mean values ± SE, and a student's t-test was performed to compare the effects of the organic and conventional cultivation systems.

## Appendix

The authors declare that they have no known competing financial interests or personal relationships that could have appeared to influence the work reported in this paper.
